# A case report: Multiple synchronous pain full glomus tumors in adjacent fingers; a review of multiple glomus tumors

**DOI:** 10.1016/j.ijscr.2024.110724

**Published:** 2024-12-10

**Authors:** Farid Najd Mazhar, Omid Mahmoudi Nasab

**Affiliations:** Bone and Joint Reconstruction Research Center, Department of Orthopedics, School of Medicine, Iran University of Medical Sciences, Tehran, Iran

**Keywords:** Glomus tumor, Glomangioma, Glomangiomatosis, Neoplasms

## Abstract

**Introduction:**

Glomus tumors (GTs) are uncommon tumors that often appear as a single lesion in the subungual region. Multiple GT is a rare clinical entity that can be associated with other diseases or in the context of genetic mutations. However, the occurrence of solitary GTs simultaneously has also been reported rarely.

**Presentation of case:**

A 37-year-old lady with a history of cold sensitivity reported having pain in her right middle and ring fingers for several years. All paraclinical examinations were normal, and the patient underwent surgery based on clinical findings suggestive of a possible diagnosis of a GT, and the patient's pain was entirely resolved following tumor excision.

**Discussion:**

GT should always be included in the differential diagnosis for multiple painful lesions.

**Conclusion:**

Early diagnosis and appropriate treatment of a GT can prevent bothersome chronic pain for the patient.

## Highlights

• A solitary glomus tumor can present multiple and simultaneously.
• In chronic pain with an unknown origin, especially in the limbs, a glomus tumor should always be among the differential diagnoses.
• Imaging studies may not reveal any findings for a glomus tumor.
• In multiple glomus tumors, it is important to consider their association with neurofibromatosis type 1.

## 1 Introduction

Glomus tumors (GTs) are rare benign tumors originating from glomus bodies commonly seen in the hand. These tumors usually appear as solitary lesions and are found subungual [1]. In 10 % of cases, the lesions can appear multiple with different presentations. Solitary cases are more commonly seen in adults in their 3rd to 5th decades of life, while multiple cases are typically observed in children, usually between 10 and 15 years earlier than solitary cases [1,2]. Multiple cases are often associated with a genetic disorder or a family history, or in conjunction with other disorders, and their treatment and outcomes can vary [3]. Pain with varying focal points in multiple GTs can lead to confusion, potentially resulting in the misdiagnosis of systemic or psychological disorders. This misinterpretation can further delay diagnosis and complicate treatment strategies. However, there have been limited reports of solitary lesions appearing in multiple and simultaneous forms, in the same or adjacent fingers, which, after accurate diagnosis, have shown improvement with treatment similar to that of solitary lesions [[4], [5], [6], [7], [8], [9], [10], [11]].

We reported a case where the patient's symptoms and lesions appeared simultaneously in two adjacent fingers, and based on our knowledge, only two similar cases have been reported previously [4,5].

## 2 Presentation of case

**Fig. 1:**
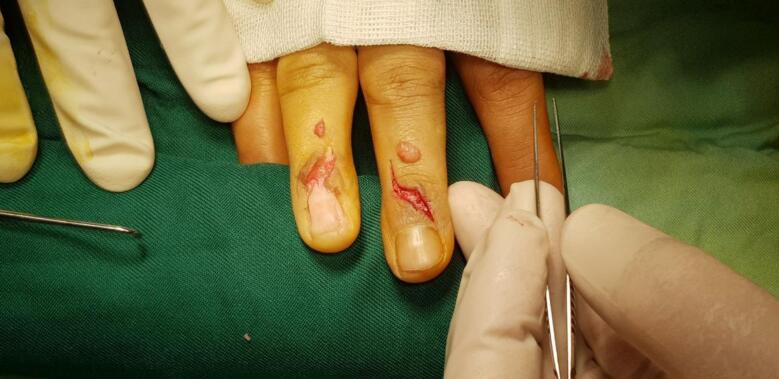
Shows the dorsal incisions in the middle and the ring finger were given at the patient's maximum tenderness points.

**Fig. 2:**
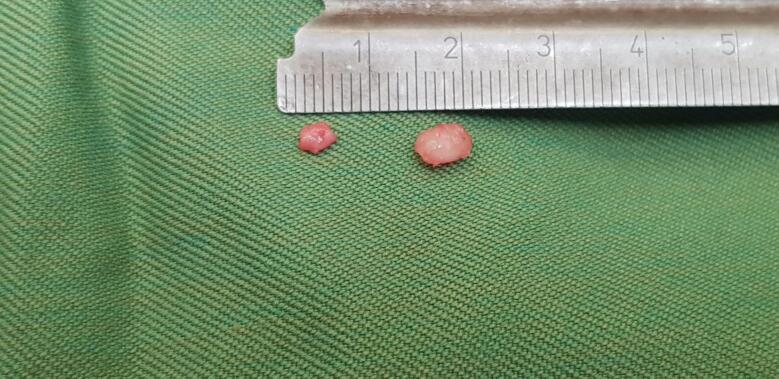
Shows tumors that were excised from the middle and the ring finger.

The patient was a 37-year-old woman who was referred to our clinic with complaints of continuous pain in the middle and ring fingers of her right hand. Three years ago, the pain began simultaneously and intensified with touch and cold. She reported no history of finger trauma. She had no underlying medical issues and mentioned no major family history. The patient's discomfort had worsened during the previous five months, prompting her to visit several clinics and undergo a variety of paraclinical examinations, such as radiography, CT scan, MRI, EMG-NCV, bone scintigraphy, and laboratory testing. Further than a minor cystic lesion in the head of the third metacarpal, there were no further abnormalities on MRI and other studies. The physical examination revealed a tiny protrusion on the dorsum of the distal phalanx, just proximal to the middle finger's nail. There was no obvious discoloration. Pin Prink's test yielded positive results on both fingers. The clinical data clearly suggested GT, so the patient underwent surgery. A lazy S incision was formed on the dorsal surface of the fingers using a digital block and tourniquet, and the ring finger's nail plate was carefully removed from its bed for easier access (Fig. 1). Tumors were identified and removed from both fingers. (Fig. 2), The ring finger nail bed did not require repair; thus the nail was relocated. Finger movements began immediately after surgery, and the patient was pleased with the pain relief at his first post-surgical appointment. Histopathological examination confirmed the presence of glomus tumors in both lesions. The patient's pain was completely relieved after surgery, and there were no signs of recurrence up to six months later. During the most recent visit, the patient mentioned her improved feeling of working with her hands, particularly touching cold objects, which had previously been a nightmare for him.

This work has been reported as being in line with the SCARE criteria [12].

## 3 Discussion

The main histopathological components of GTs include glomus cells, vascular structures, and smooth muscle tissue. GTs are classified into three subtypes based on proportional component dominance: GT or solid type (the most common variety), glomangioma, and glomangiomyoma. GTs are categorized into two categories based on their clinical presentation: solitary and multiple [13]. GTs appear as a solitary type in around 90 % of cases [1]. Because of the amount and abundance of glomus bodies in the fingers, particularly in the subungual region, these are the most common locations for tumor development. Given the 75 % incidence of musculoskeletal GTs in the hand, these tumors are classified into two types: digital and extradigital, according to their place of occurrence. However, GTs can appear in any organ and tissues [1,2].

Multiple GTs may indicate a separate condition requiring specific attention. Clinically, this multifocal type may not show the characteristic symptoms associated with GTs and is more commonly seen in the trunk and upper extremities. Multifocality has been linked to a number of circumstances, including pediatric presentation, familial inheritance, and pregnancy [[1], [2], [3]].

On rare occasions, solitary lesions presenting with the classic symptoms of this tumor may manifest as multiple lesions simultaneously in typical locations. Although the management and outcomes of these cases are generally comparable to those of solitary tumors, the presence of asymptomatic lesions at the time of surgery and the diversity of lesions may result in treatment failure or an increased risk of recurrence because of the persistence of these lesions. [[4], [5], [6], [7], [8], [9], [10], [11]]

Graham and his colleagues described a similar instance in the ring and middle fingers of one hand, however, unlike our case, both lesions were at the nail beds and the patient's symptoms emerged first on one finger and then on the other. [4] Lee and colleagues described a case in which the patient's symptoms were present in eight fingers on both hands, and after surgery, the diagnosis of GT was confirmed in five fingers. [5] Rashid and Remington observed further examples in non-adjacent fingers, where the lesions were asynchronous and situated in the periungual area or nail beds. [8,10] None of the patients in the publications evaluated, including ours, showed signs of tumor recurrence. As a result, it is possible to postulate that the presence of a solitary glomus tumor in multiple locations in the fingers does not constitute a risk factor for eventual tumor recurrence, as long as treatment is performed effectively. Clinical symptoms, as reported in many other studies, play an important role in detecting this type of tumor and can help avoid unnecessary paraclinical evaluations. The presence of this tumor in multiple forms might also be accompanied by the usual signs of this tumor, such as local tenderness and sensitivity to cold. In questionable circumstances, paraclinical techniques such as MRI or ultrasound can be useful. However, even a capable method for diagnosing soft tissue tumors like MRI may also be non-diagnostic, as in our case. About 10 % of cases may remain undetected by MRI, and MRI findings are not specific to this tumor. Furthermore, using the appropriate techniques and slice thickness is critical when evaluating small lesions. Despite these limitations, MRI plays a significant role in determining the exact lesion's location and evaluating its extent. [14]

Multiple hereditary GTs, also known as familial glomangiomas, are venous malformations that contain glomus cells. Despite infrequent cases, it is a hereditary condition caused by a mutation in the glomulin gene (GLMN), with autosomal dominant inheritance in most cases [3]. Unlike the solitary variation, these lesions are more commonly seen in young people and have an equal occurrence in both sexes. The majority of instances occur in the extremities and are typically less painful [8]. When compared to venous malformations, these lesions are pink to bluish-purple or dark blue and have a cobblestone-like appearance with mild hyperkeratosis, particularly if the lesion is located on an extremity. Unlike venous malformations, these lesions cannot be drained by applying external pressure to them, which will cause pain [15].

Glomangiomatosis (also known as diffuse glomus tumor) represents a rare subtype of GT, accounting for approximately 5 % of reported cases [16]. Glomangiomatosis (also known as diffuse glomus tumor) is an uncommon form of GT that accounts for about 5 % of reported cases [16]. The World Health Organization defines this condition as “…an extremely rare subtype of glomus tumor with an overall architectural resemblance to a diffuse vascular malformation… but containing nests of glomus cells investing walls” [17]. This illness is distinguished by multiple, extensive, and painful lesions that predominantly afflict young people and are often located on the distal extremities. While lesions are more common in the hand, they differ from conventional glomus tumors in that they are not subungual and tend to be larger and deeper. [16]. The lesions are categorized as intermediate lesions because, in spite of their aggressive and infiltrative nature, they lack the morphological characteristics usually associated with malignancy. Because of their about 10 % chance of recurrence after surgery, these lesions require further postoperative monitoring [[16], [17], [18], [19]].

Another condition associated with the potential for multiple GTs is neurofibromatosis type 1 (NF1), which occurs in approximately 30 % of cases. Recent studies have established a strong correlation between this disease and GTs, leading to the classification of GTs as part of the spectrum of tumors associated with NF1 [2,3]. Since neurofibromatosis is generally diagnosed in early childhood, the chance of encountering an undiagnosed case in an adult with a GT is minimal. In children, a GT can indicate the presence of this disease, necessitating a more thorough examination for additional symptoms related to the condition [2,3,20].

## 4 Conclusion

The diagnosis of GTs has always been challenging, and it may take up to a decade for a definitive diagnosis, during which the patient undergoes multiple examinations and various paraclinical studies. In multiple glomus cases, this issue becomes more complex, and the patient may be treated for systemic diseases such as Raynaud's syndrome or psychiatric disorders, leading to catastrophic conditions. Therefore, obtaining a detailed medical history, thorough examinations, and attention to the possibility of multiple and simultaneous lesions can be very helpful. The association of multiple lesions with other diseases should be considered, especially when accompanied by atypical manifestations, such as in childhood. Similar to solitary cases, surgical treatment is thought to be the most effective treatment option in multiple GTs.

## Statement

During the preparation of this work, the authors used AI to improve readability and language. After using this service, the authors reviewed and edited the content as needed and took full responsibility for the content of the publication.

## Informed consent

Written informed consent was obtained from the patient to publish this case report and accompanying images.

## Ethical approval

There is no need for approval from an ethics committee for this type of article (case report).

## Guarantor

Omid Mahmoudi Nasab guarantor for this case report.

Bone and Joint Reconstruction Research Center, Department of Orthopedics, School of Medicine, Iran University of Medical Sciences, Tehran, Iran

Email Address: o.mahmoodinasab@gmail.com

Mobile phone number: +989165893626

ORCID: 0000-0001-9982-3581

## Funding

This research did not receive any grant from funding agencies in the public, commercial, or non-profit sectors.

## Declaration of competing interest

The authors declared no conflicts of interest.
